# Iron Oxide Particles Alter Bacterial Uptake and the LPS-Induced Inflammatory Response in Macrophages

**DOI:** 10.3390/ijerph18010146

**Published:** 2020-12-28

**Authors:** Lewis J. Williams, Stephen G. Tristram, Graeme R. Zosky

**Affiliations:** 1 Tasmanian School of Medicine, University of Tasmania, 7000 Hobart, Australia; Lewis.Williams@utas.edu.au; 2 School of Health Sciences, University of Tasmania, 7250 Launceston, Australia; Stephen.Tristram@utas.edu.au; 3 Menzies Institute for Medical Research, University of Tasmania, 7000 Hobart, Australia

**Keywords:** geogenic, particulate matter, macrophages, inflammatory cytokines, NTHi

## Abstract

Exposure to geogenic (earth-derived) particulate matter (PM) is linked to severe bacterial infections in Australian Aboriginal communities. Experimental studies have shown that the concentration of iron in geogenic PM is associated with the magnitude of respiratory health effects, however, the mechanism is unclear. We investigated the effect of silica and iron oxide on the inflammatory response and bacterial phagocytosis in macrophages. THP-1 and peripheral blood mononuclear cell-derived macrophages were exposed to iron oxide (haematite or magnetite) or silica PM with or without exposure to lipopolysaccharide. Cytotoxicity and inflammation were assessed by LDH assay and ELISA respectively. The uptake of non-typeable *Haemophilus influenzae* by macrophages was quantified by flow cytometry. Iron oxide increased IL-8 production while silica also induced significant production of IL-1β. Both iron oxide and silica enhanced LPS-induced production of TNF-α, IL-1β, IL-6 and IL-8 in THP-1 cells with most of these responses replicated in PBMCs. While silica had no effect on NTHi phagocytosis, iron oxide significantly impaired this response. These data suggest that geogenic particles, particularly iron oxide PM, cause inflammatory cytokine production in macrophages and impair bacterial phagocytosis. These responses do not appear to be linked. This provides a possible mechanism for the link between exposure to these particles and severe bacterial infection.

## 1. Introduction

Bronchiectasis is a chronic obstructive lung disease that develops as a result of prolonged inflammation leading to structural changes in the airways and recurrent infections [1]. The prevalence of bronchiectasis is almost three times higher in Australian Aboriginals living in rural communities [2] and these communities have among the highest prevalence of bronchiectasis in children globally [3]. While these communities often have comparatively higher rates of smoking and restricted access to medical services [4,5,6], other environmental factors may be contributing to this disease disparity [7,8,9]. Previous studies have suggested that exposure to air pollution is associated with increased bronchiectasis exacerbations and mortality [10,11]. While Aboriginal communities are typically exposed to lower levels of traffic-derived pollution than urban centres, they are exposed to high concentrations of geogenic (earth-derived) particulates which consist primarily of iron oxide and silica [5,7]. The potential contribution of these airborne particles to the burden of bronchiectasis is unclear.

Non-typeable *Haemophilus influenzae* (NTHi) is the most common pathogen isolated from individuals with bronchiectasis [2,12,13,14,15]. We have previously shown that exposure to geogenic particles increases viral susceptibility in vivo [16] and has the capacity to alter NTHi growth in vitro [17]. However, the impact of these particles on the cellular response to NTHi is largely unknown.

Given the critical role of macrophages in the response to inhaled particulates [18,19] and pathogenic bacteria [20], we assessed the production of inflammatory cytokines in macrophages in response to iron oxide particles alone, or in combination with silica, with or without prior exposure to bacterial endotoxin. We also investigated the effect of the particles on the phagocytic capacity of macrophages to NTHi and potential mechanisms explaining the differences we observed. 

## 2. Materials and Methods

### 2.1. Particle Preparation

Haematite (Fe_2_O_3_ 310050; Sigma-Aldrich, St. Louis, MO, USA), magnetite (Fe_3_O_4_ 310069; Sigma-Aldrich, St. Louis, MO, USA) and α-quartz (SiO_2_ 1878B; National Institute of Standards and Technology, Gaithersburg, MD, USA) particles (<5 µm) were used for cell exposure experiments. Haematite and magnetite were chosen as common forms of naturally occurring iron oxide with different redox potentials (Fe II and Fe III respectively) which may impact on the cellular response (26). Particles were exposed to UV light for two hours prior to experimentation and washed with centrifugations and saline to remove any bacterial contamination. Particle samples were dispersed in RPMI-1640 media and vortexed thoroughly for 30 s to ensure even distribution of particles within the suspension before application to the cells.

### 2.2. Cell Culture

The human leukaemic monocyte cell line, THP-1 (Sigma-Aldrich, St. Louis, MO, USA), was cultured in 25 cm^2^ flasks (CLS430639; Corning Inc., Corning, NY, USA), using RPMI-1640 (30-2001; American Type Culture Collection, Manassas, VA, USA) supplemented with 10% foetal bovine serum (FBS; Gibco A31605, Life Technologies, Carlsbad, CA, USA) and 1% penicillin-streptomycin (P4333; Sigma-Aldrich, St. Louis, MO, USA) at 37 °C in a humidified atmosphere of 5% CO_2_.

### 2.3. Peripheral Blood Mononuclear Cell (PBMC) Isolation and Culture

The following collection of human blood has been approved by the Tasmanian Health and Medical Human Research Ethics Committee (H0016505). Peripheral blood mononuclear cells (PMBCs) were isolated from six healthy adults aged between 20 and 50 years of age. Briefly, 32 mL of blood was collected from each patient using EDTA-BD Vacutainers (366643; Becton Dickinson, Franklin Lakes, NJ, USA). Samples were incubated at room temperature for 20 min with gentle mixing. Blood was aseptically layered onto 4 mL of Histopaque (10771; Sigma-Aldrich, St. Louis, MO, USA) in a 1:1 ratio before being centrifuged at 100 g for 30 min. Density gradient separation allowed the isolation of mononuclear cells which were then washed twice by centrifugation at 300 g. Cells were resuspended in serum-free RPMI-1640 and seeded at a density of 6 × 10^6^ cells/mL. Cells were incubated for 1 h at 37 °C in a humidified atmosphere of 5% CO_2_ and then washed twice to remove non-adherent cells, providing pure monocyte cultures. RPMI-1640 media containing 10% FBS was supplemented with 2 ng/mL of granulocyte-macrophage colony-stimulating factor (GM-CSF 300-03; Pepro Tech Inc., Rocky Hill, NJ, USA) for 12 days, refreshing every 3 days.

### 2.4. Cell Exposure Trials

Cells were suspended in RPMI-1640 with 10% FBS and seeded into 12-well plates (CLS3512; Corning Inc., Corning, NY, USA) at a concentration of 6 × 10^5^ cells/well. THP-1 cells were differentiated into macrophages by exposure to 25 µM of Phorbol 12-myristate 13-acetate (PMA P1585; Sigma-Aldrich, St. Louis, MO, USA) for 48 h, followed by recovery in PMA-free growth media for 24 h. Cells were then either exposed to 10 ng/mL *E. Coli*-derived lipopolysaccharide (LPS L4391; Sigma-Aldrich, St. Louis, MO, USA) or LPS-free media for 4 h before particle exposure. To investigate the dose-dependent effects of haematite, magnetite and silica individually, cells were exposed to 0, 10, 25 or 50 μg/mL of each particle type. Concentrations were chosen to be consistent with similar PM toxicology studies (30–35). Cytotoxicity and the production of interleukin (IL)-1β, IL-6, IL-8 and tumour necrosis factor-α (TNF–α) were assessed after 24 h of particulate exposure. The individual particle effects were established before assessing the impact of silica and iron, in combination, on the response. Cells were exposed to a 2:1 silica: iron ratio, which reflects the proportion of these elements in real-world particles (12). The total dose used (50 µg/mL) was chosen based on the initial experiments, to reflect a concentration that would not cause large levels of cytotoxicity but was sufficient to produce a cytokine response. Cytotoxicity and cytokine production were assessed after 24 h of exposure to particulates as described previously. 

Subsequent bacterial internalisation experiments used the same 50 µg/mL particle concentration for 24 h. All experiments were repeated in six independent trials. Each trial was conducted on a different day using fresh cell cultures and reagents including particles and LPS preparations to allow valid statistical comparisons between exposure groups.

### 2.5. Cytotoxicity

The LDH assay (G1780; Promega Corporation, Madison, WI, USA) was used as a marker of cytotoxicity. LDH levels were measured according to the manufacturer’s instructions. Absorbance was read with the Spectra Max M2 plate-reader (Molecular Devices, San Jose, CA, USA).

### 2.6. Inflammatory Cytokine Production

Levels of human interleukin-1β (IL–1β; R&D Systems DY201), interleukin-6 (IL–6; R&D Systems DY206), interleukin-8 (IL–8; R&D Systems DY208) and tumour necrosis factor-α (TNF–α; R&D Systems DY210) in the cell supernatant 24 h post-exposure to the particles were assessed by enzyme-linked immunosorbent assay (ELISA) according to the manufacturer’s instructions. The minimum detection limits for IL–1β, IL–6, IL-8 and TNF–α were 7.81, 9.38, 31.3 and 15.6 pg/mL respectively. Absorbance was read with the Spectra Max M2 plate-reader (Molecular Devices, San Jose, CA, USA).

### 2.7. Bacterial Isolates and Culture

Eight clinical pathogenic strains of NTHi were obtained from the University of Tasmania Culture Collection and identity was confirmed by genetic markers. Bacteria were cultured on blood agar and transferred to a brain heart infusion broth overnight before exposure to cells. Bacteria were cultured on an orbital shaker at 200 rpm at 37 °C in atmospheric CO_2_.

### 2.8. Bacterial Exposure

Bacterial broth suspensions were standardised to an optical density of 0.6 at 600 nm (OD_600_). From this, 1 mL of each bacterial suspension was centrifuged at 6000 g for 10 min before three washes in sterile-filtered phosphate-buffered saline (PBS). Bacteria were resuspended in a total of 1 mL of PBS each and fluorescently tagged with 1 µL CellTrace Far Red (C34564; Thermo Fisher Scientific, Waltham, MA, USA) for 20 min at room temperature in the absence of light. Bacteria were diluted to a 1:30 concentration before 30 µL of bacterial suspension was applied per well in a 96-well plate and incubated for 3 h at 37 °C and 5% CO_2_.

After bacterial exposures, samples were washed using PBS and exposed to their respective growth media supplemented with 200 µg/mL of gentamicin to remove remaining extracellular bacteria. Samples were incubated for 4 h at 37 °C and 5% CO_2_. Following PBS washing, trypsin detached cell samples were made to a final volume of 200 µL/ well for flow cytometry.

### 2.9. Flow Cytometry

Fluorescent staining was analysed using a BD Facscanto II flow cytometer (BD Biosciences, San Jose, CA, USA). Data were analysed using FCS Express 6 (DeNovo software, Pasadena, CA, USA), using fluorescence minus one (FMO) controls to determine gating strategies. Compensation was performed using single colour stained cells, and compensation matrices were calculated and applied. Cell populations were distinguished based on forward scatter (FSC) height and width (see Supplementary Materials Figure S1). Gating strategies mitigated the presence of any remaining extracellular bacteria, while intracellular bacteria were quantified using the median fluorescent intensity. 

### 2.10. Statistical Analysis

Comparisons between groups were made using repeated measures one-, and two-way ANOVA. When significance was determined for the main factors by ANOVA, the Holm–Sidak post-hoc test was used to examine individual between group differences. Where necessary, the data were log transformed to satisfy the assumptions of normal distribution of the error terms and homoscedasticity of the variance. All data are presented as mean (SD) and values of *p* < 0.05 were considered statistically significant. All statistical analyses were conducted using SigmaPlot (v12.5 Systat, San Jose, CA, USA). 

## 3. Results

### 3.1. Effect of Silica and Iron Oxide PM on Inflammation

#### 3.1.1. Cytotoxicity

**Figure 1:**
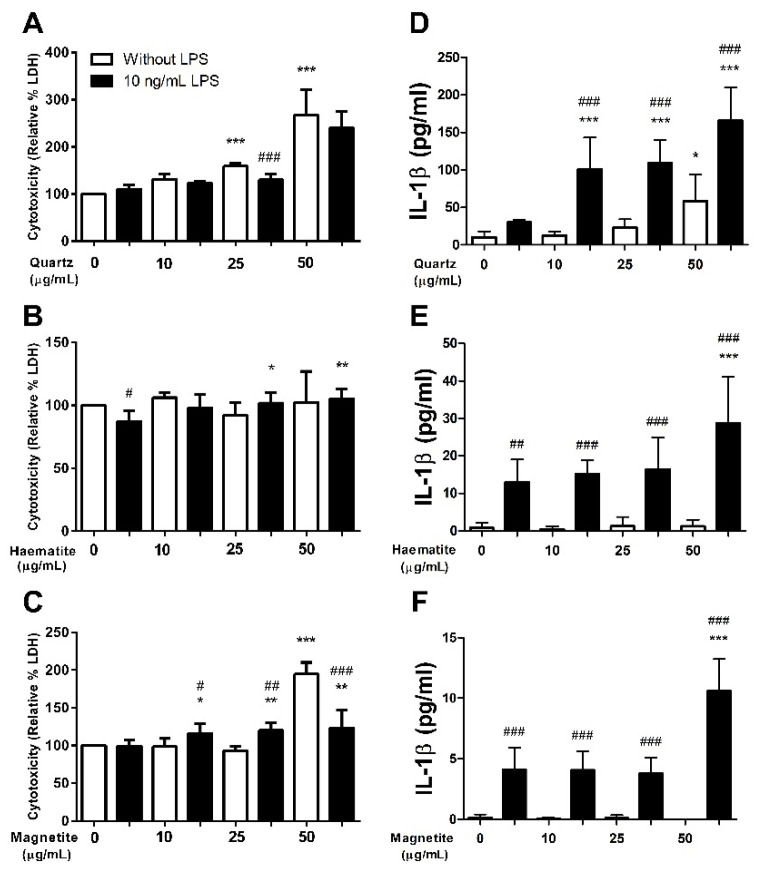
Lactate dehydrogenase (LDH; **A**–**C**) and IL-1β (**D**–**F**) levels in the supernatant of THP-1-derived macrophages exposed to quartz (**A**,**D**), haematite (**B**,**E**) or magnetite (**C**,**F**) for 24 h, with (black bars) or without (white bars) 4 h of prior lipopolysaccharide (LPS) exposure. LDH data are represented as a relative percentage increase in optical density value compared to the control (100%). Data are presented as mean (SD) from 6 independent experiments. *, ** and *** indicate *p* < 0.05, *p* < 0.01 and *p* < 0.001 respectively versus control. #, ## and ### indicate *p* < 0.05, *p* < 0.01 and *p* < 0.001 respectively for LPS vs no LPS at the given dose.

Quartz caused a dose-dependent increase in LDH production, with an increase observed at the 25 μg/mL concentration (*p* < 0.001) and a further increase at 50 μg/mL (*p* < 0.001) (Figure 1A). Exposure to LPS reduced the quartz-induced LDH production, such that an increase in LDH was only detectable in response to the 50 μg/mL dose (*p* < 0.001), and the LDH production at this dose was significantly lower than that of the cells exposed to the same dose of quartz without LPS (*p* < 0.001) (Figure 1A). Exposure to haematite had no effect on LDH levels (*p* > 0.05 for all doses) (Figure 1B). In the haematite trials, LPS caused a small but statistically significant decrease in LDH production (*p* = 0.02) (Figure 1B). In contrast, when cells were exposed to LPS in combination with haematite there was a small increase in LDH production at the 25 μg/mL dose (*p* = 0.04) that did not increase further with exposure to 50 μg/mL (*p* = 0.74) (Figure 1B). Exposure to magnetite also increased LDH production at the 50 μg/mL dose (*p* < 0.001) (Figure 1C). Exposure to LPS changed the LDH-magnetite dose response relationship such that the increase in LDH production was detectable at 25 μg/mL (*p* = 0.04) but did not reach the same level at 50 μg/mL as that seen in cells exposed to magnetite without LPS (*p* < 0.001) (Figure 1C).

#### 3.1.2. Interleukin-1β

Quartz induced a dose-dependent increase in interleukin-1β (IL-1β) levels at 50 µg/mL (*p* = 0.03) (Figure 1D). LPS significantly augmented this response (*p* < 0.001 for all doses) (Figure 1D). In contrast, haematite exposure did not alter IL-1β levels (*p* > 0.05 for all doses) (Figure 1E). However, LPS resulted in significant increases in IL-1β expression at all concentrations (*p* < 0.003) (Figure 1E). This response was augmented by exposure to haematite with the highest IL-1β observed at 50 µg/mL (*p* < 0.001) (Figure 1E). Similarly, magnetite alone had no effect on IL-1β production (*p* > 0.05 for all doses) (Figure 1F) but did enhance LPS-induced production of IL-1β with the highest expression observed at 50 µg/mL (*p* < 0.001) (Figure 1F).

#### 3.1.3. Interleukin-6

**Figure 2:**
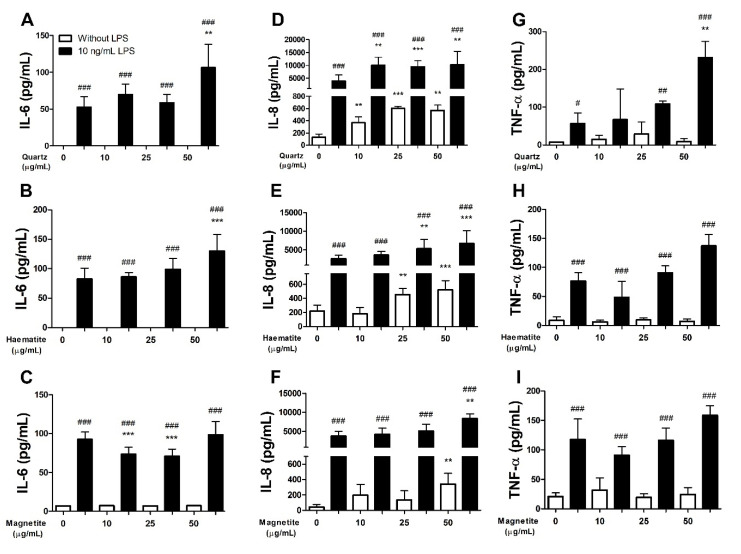
IL-1β (**A**–**C**), IL-8 (**D**–**F**) and TNF-α (**G**–**I**) levels in the supernatant of THP-1-derived macrophages exposed to quartz (**A**,**D**,**G**), haematite (**B**,**E**,**H**) or magnetite (**C**,**F**,**I**) for 24 h, with (black bars) or without (white bars) 4 h of prior lipopolysaccharide (LPS) exposure. Data are presented as mean (SD) from 6 independent experiments. ** and *** indicate *p* < 0.01 and *p* < 0.001 respectively versus control. #, ## and ### indicate *p* < 0.05, *p* < 0.01 and *p* < 0.001 respectively for LPS vs no LPS at the given dose.

Neither quartz (Figure 2A), haematite (Figure 2B) nor magnetite (Figure 2C) exposure caused any significant change in interleukin-6 (IL-6) production (*p* > 0.05 for all doses) (Figure 2). LPS significantly increased IL-6 production (*p* < 0.001 for all doses) (Figure 2). Exposure to 50 µg/mL of quartz or haematite significantly enhanced the LPS-induced IL-6 production (quartz, *p* = 0.006; haematite, *p* < 0.001) (Figure 2A,B). In contrast, magnetite did not enhance the IL-6 response induced by LPS (Figure 2C). 

#### 3.1.4. Interleukin-8

Quartz induced an increase in interleukin-8 (IL-8) production (Figure 2D) which was detectable at 10 µg/mL (*p* = 0.004) and did not increase further at higher particle doses (*p* > 0.05) (Figure 2D). LPS caused a profound effect on IL-8 production (*p* < 0.001 for all doses) that was further enhanced by exposure to 10 µg/mL of quartz (*p* = 0.004) but did not increase further with higher doses of particles (*p* > 0.75) (Figure 2D). Haematite induced a dose-dependent increase in IL-8 which was detectable at 25 µg/mL (*p* = 0.002) with no further increase at 50 µg/mL (*p* = 0.56) (Figure 2E). Exposure to haematite particles enhanced LPS-induced IL-8 production resulting in a similar dose-dependent response to that observed without LPS (Figure 2E). The same pattern was observed in response to magnetite, although the increase in IL-8 with (*p* = 0.002) and without (*p* = 0.002) LPS was only observed at the 50 µg/mL particle dose (Figure 2F).

#### 3.1.5. Tumour Necrosis Factor-α

Tumour necrosis factor-α (TNF-α) expression was not altered in response to quartz (Figure 2G), haematite (Figure 2H) or magnetite (Figure 2I) (*p* > 0.05 for all doses). LPS exposure increased the production of TNF- α which was enhanced further by exposure to 25 (*p* = 0.010) and 50 µg/mL (*p* < 0.001) of quartz particles (Figure 2G). In contrast, haematite (*p* > 0.09) and magnetite (*p* = 0.146) did not modify the LPS-induced increase in TNF-α production (Figure 2H,I).

### 3.2. The Effect of Particles on NTHi Phagocytosis

**Figure 3:**
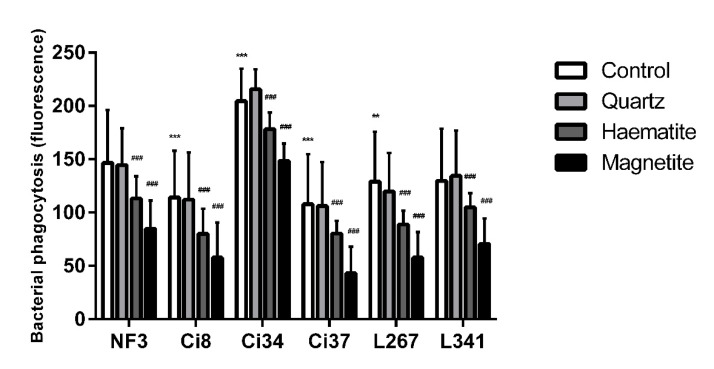
Relative phagocytosis of six NTHi isolates in THP-1-derived macrophage exposed to 0 or 50 µg/mL of quartz, haematite or magnetite or 24 h. Data are presented as mean (SD) from 6 independent experiments. ** and *** indicate *p* < 0.01 and *p* < 0.001 respectively versus NF3 control. ### indicates *p* < 0.001 versus particle control within each isolate.

The effect of particle exposure on NTHi phagocytosis was assessed in six clinical isolates (Ci8, Ci34, Ci37, L267, L341 and NF3) [21]. There were variations in baseline levels of phagocytosis of the different isolates with higher phagocytosis of the Ci34 strain (*p* < 0.001) and lower levels of phagocytosis of the Ci37, Ci8 (*p* < 0.001) and L267 (*p* = 0.003) strains compared to the reference strain (NF3) (Figure 3). Compared to baseline levels of phagocytosis, haematite (*p* = 0.013) and magnetite (*p* < 0.001) decreased the internalisation of all strains of bacteria while quartz had no effect (*p* = 0.989). The effect of magnetite was greater than the effect of haematite (*p* = 0.010) (Figure 3).

### 3.3. Combined Effect of Quartz and Iron Oxide

Having established the effect of iron oxide, and silica, particles alone on the response, we also assessed whether the combination of iron oxide and silica modified the response. We found that the combination exposure did not modify the individual responses we observed in terms of cytotoxicity, cytokine production or NTHi phagocytosis (*data not shown*). 

### 3.4. Responses in PBMCs

#### 3.4.1. Cytotoxicity

Quartz induced significant cytotoxicity when compared to control (*p* = 0.001), whereas haematite and magnetite (*p* > 0.47) had no cytotoxic effects in PBMCs (see Supplementary Materials Figure S2).

#### 3.4.2. Cytokine Production

**Figure 4:**
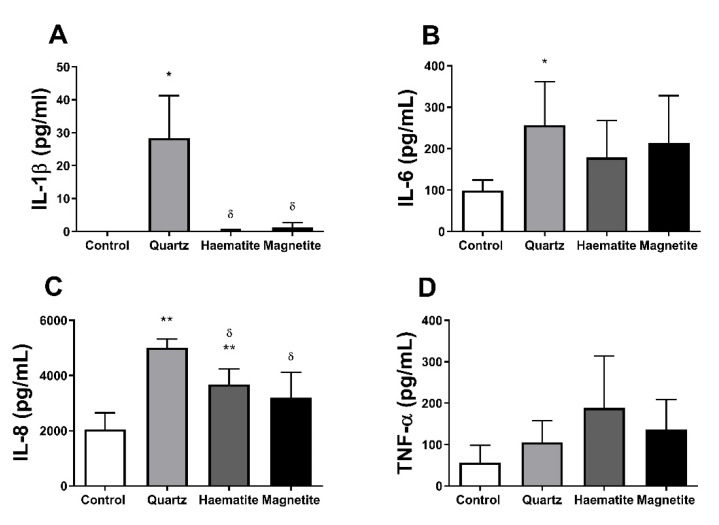
Interleukin (IL)-1β (**A**), IL-6 (**B**), IL-8 (**C**) and tumour necrosis factor (TNF)-α (**D**) levels in the supernatant of peripheral blood mononuclear cell (PBMC)-derived macrophages exposed to 0 or 50 µg/mL of quartz, haematite or magnetite for 24 h. Data are presented as mean (SD) from 6 independent experiments. * and ** indicate *p* < 0.05 and *p* < 0.01 respectively versus control and δ indicates *p* < 0.05 versus quartz.

Similar to the response observed in THP-1 macrophages, only quartz induced IL-1β production compared to control (Figure 4A, *p* = 0.017) in PBMCs. Haematite and magnetite (*p* > 0.18) had no effect on IL-1β compared to control (*p* > 0.185). Only quartz increased IL-6 expression compared to control (Figure 4B, *p* = 0.047). Conversely, both quartz (Figure 4C, *p* = 0.001) and haematite (*p* = 0.003), but not magnetite (*p* = 0.125), increased IL-8 production when compared to control. However, the effect of quartz was higher than haematite (*p* = 0.012). There was no effect of any exposure on TNF-α production (Figure 4D, *p* > 0.281).

#### 3.4.3. NTHi Phagocytosis

**Figure 5:**
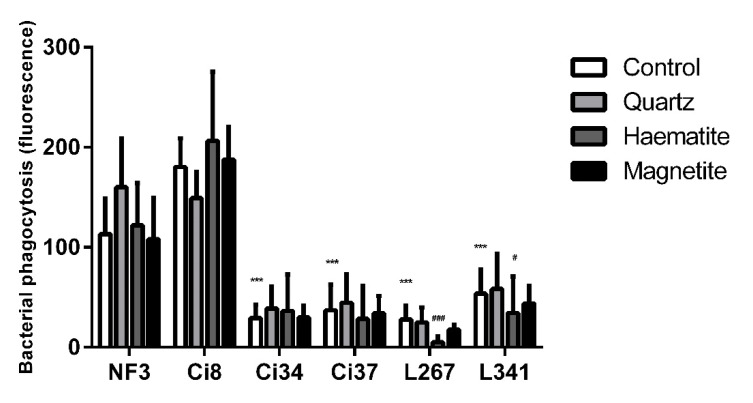
Relative phagocytosis of six NTHi isolates in PBMC-derived macrophage exposed to 0 or 50 µg/mL of quartz, haematite or magnetite or 24 h. Data are presented as mean (SD) from 6 independent experiments. *** indicate *p* < 0.001 versus NF3 control. # and ### indicate *p* < 0.05 and *p* < 0.001 respectively versus particle control within each isolate.

Like the THP-1 response, there were differences between NTHi strains (Figure 5; *p* < 0.05); however, the effect of particles on the response on PBMCs was different to that observed in THP-1 cells. In PBMCs, isolates L267 and L341 were phagocytosed less after exposure to haematite (*p* < 0.001 and *p* = 0.028 respectively), similar to observations of THP-1-derived macrophages. In contrast to the THP-1 data, there were no effects of particle exposure on the phagocytosis of the other isolates (Figure 5).

## 4. Discussion

Regional Australian Aboriginal communities are exposed to high levels of geogenic particulate matter (Shepherd et al., 2019), dominated by silica and iron oxides (Zosky et al., 2014). These communities also have a disproportionate burden of chronic respiratory infections resulting in bronchiectasis (Blackall et al., 2018). The present study aimed to investigate the effect of iron oxide, with or without prior LPS exposure, on inflammation in macrophages and on bacterial phagocytosis as potential contributors to the detrimental health outcomes observed in individuals exposed to iron-laden geogenic PM [9]. Iron oxide particles clearly modified the effect of LPS on the macrophages, which varied between particle types and across cytokines. While haematite and magnetite had no effect on IL-1β production, they both enhanced LPS-induced IL-1β production in THP-1 cells. Similarly, quartz and haematite, but not magnetite, enhanced LPS-induced IL-6 production while all three particle types enhanced LPS-induced IL-8 in these cells. Similar responses to these particles were observed in PBMCs. Both haematite and magnetite, but not quartz, significantly decreased NTHi internalisation in THP-1 cells. However, only the effects of haematite were confirmed in PBMCs. Based on these observations, the previously observed link between exposure to iron-laden geogenic particles and poor respiratory health may be explained by the inflammatory effect on macrophages and the impairment of NTHi internalisation by these cells. The magnitude of these effects is likely to vary depending on the chemical composition of the inhaled particles (e.g., quartz vs haematite vs magnetite), whether there is a prior inflammatory stimulus present (e.g., infection) and the strain of NTHi present. 

LPS is a component of the outer membrane of Gram-negative bacteria and is central to mediating the inflammatory response via binding to specific host receptors [22]. LPS binds to toll-like receptor (TLR)-4 [23], leading to the upregulation of NF-κB, and enhanced transcription of a range of pro-inflammatory genes including IL-1β, IL-6, IL-8 and TNF-α [24,25,26]. Accordingly, we found that LPS upregulated the expression of all the inflammatory cytokines we measured. The most overt difference between the effect of silica and iron oxide exposures was the release of IL-1β by quartz in absence of LPS. This effect of silica is consistent with results of previous studies and is likely to be driven by NF-κB-independent NOD-like receptor pyrin domain-containing 3 (NLRP-3) inflammasome activation [27,28,29,30]. It is worth noting the observed effect of quartz on cell cytotoxicity at the highest dose. This will have impacted on the capacity of the cells to produce cytokines, meaning that the relative increase in IL-8 and IL-1β production in response to quartz is likely to be underestimated. The absence of an effect of iron oxide on the IL-1β production suggests that iron oxide is unable to activate this same pathway. While iron oxide nanoparticles can induce some level of IL-1β, due to partial NLRP-3 activation, this is highly dependent on the shape of the particles [31], whereby spherical iron oxide particles, which have a similar morphology to the particles used in the present study [32], caused a minimal response in this pathway. The particles used in the present study were all in the < 5µm size range [32], which is consistent with the particle size observed in community sampled geogenic PM [33].

IL-8 is a neutrophil chemoattractant protein which can also be upregulated via NF-κB in response to LPS [34]. Our data showed that, in the absence of LPS, IL-8 was produced in response to all particle types. In epithelial cells, silica induces IL-8 through endocytosis-independent p38/AP-1 and ERK1/2/CEBP upregulation [23,35,36,37]. While the mechanism is unclear from our experiments, given the similarities between the IL-8 responses, it is possible that iron oxide particles may also activate the AP-1/CEBP pathway. This warrants further investigation. 

IL-6 differs from the other cytokines discussed as it acts as both an anti-inflammatory mediator and macrophage activator [24]. IL-6 synthesis is initiated via multiple pathways including upregulation of NF-κB, CEBP and IL-1β [38,39,40,41,42,43,44,45]. As expected, we observe a strong LPS-induced response. We also observed a modifying effect of the particles on this response. Interestingly, unlike both quartz and haematite, magnetite had a moderate suppressive effect on LPS-induced IL-6 at lower doses. As previously mentioned, IL-6 plays an anti-inflammatory role in the lung, supressing overexpression of TLRs, including TLR-4 [46]; magnetite may have the capacity to prevent this response. We saw no effect of any particle on IL-6 production in the absence of LPS, which is surprising given the increase in IL-1β production. Taken together, these observations suggest the regulation of IL-6 production in response to particles, and the modifying effect of these particles on the LPS response, is complex [47]. 

In contrast to quartz, both magnetite and haematite reduced the phagocytosis of NTHi by THP-1 macrophages. Given that quartz typically induced higher levels of inflammation in these cells than the iron oxides, the impairment in NTHi phagocytosis seems to be uncoupled from the inflammatory response. While the mechanism is unclear from our study, macrophage dysfunction has a profound impact on airway health. For example, mice with depleted macrophages are more susceptible to experimental pneumonia due to a lack of bacterial clearance [48,49]. Accordingly, a reduction in macrophage phagocytotic capacity in commonly found in bronchiectasis and is hypothesised to pre-empt, and probably contribute to the development of, the disease [50]. This highlights the importance of macrophage function and points to a possible mechanism linking exposure to geogenic particles and an increased risk of severe bacterial infection.

It is worth noting that some of the responses we observed in THP-1 cells were not replicated in the PBMC-derived macrophages. It is possible that this is due to the method of cell transformation. THP-1 were transformed using PMA, a commonly use protocol that yields an “M0” macrophage, whereas the GM-CSF used to transform the PBMC macrophages results in an “M1” phenotype. These phenotypic differences may impact on the phagocytic potential of the cells. Nonetheless, haematite still impacted NTHi phagocytosis in some strains in the PBMCs, suggesting that the impact of iron oxide is preserved across macrophage sub-types. 

Limitations of the present study must be acknowledged. While we have investigated the effects and interactions of the two major components of geogenic PM on the cell response, silica and iron oxide do not fully represent geogenic PM in its entirety, nor do they fully represent the vast heterogeneity of naturally occurring geogenic PM. We chose to focus on silica and iron oxide as these are the two constituents that have shown the most robust correlation with negative health outcomes in previous studies [33,51]. Lastly, we utilised a commonly found cell line and transformed primary macrophages from the circulation, neither of which display the same phenotype as primary alveolar macrophages. However, the consistency of the inflammatory responses, and some of the phagocytosis response, in the different macrophage types we used suggests that it is likely that the effect of iron oxide particles we have shown is conserved across macrophages with different phenotypes.

## 5. Conclusions

The present study has confirmed the pro-inflammatory potential of inhalable quartz in macrophages, but importantly has also shown that haematite, the most common iron oxide found in the dusts affecting regional Australian Aboriginal communities, modifies the inflammation induced by a bacterial stimulus and the ability of macrophages to phagocytose NTHi. Haematite has long been viewed as a biologically inert particle due to its mild inflammatory profile. However, our results provide novel evidence that iron oxide suppresses NTHi phagocytosis which may contribute to the high burden of pathogenic respiratory NTHi infection in at-risk communities. This has important implications for all individuals exposed to iron-laden particulates.

## Data Availability

The data presented in this study are available on request from the corresponding author.

## Supplementary Materials

The following are available online at https://www.mdpi.com/1660-4601/18/1/146/s1, Figure S1: Example flow cytometry analysis of peripheral blood mononuclear cell (PBMC)-derived macrophages phagocytosing NTHi, Figure S2: Lactate dehydrogenase (LDH) in the supernatant of PBMC-derived macrophages exposed to quartz, haematite or magnetite for 24 h.
