# Supplementary material for: Iron Oxide Particles Alter Bacterial Uptake and the LPS-Induced Inflammatory Response in Macrophages
Source: Int J Environ Res Public Health. 2020 Dec 28;18(1):146. doi: 10.3390/ijerph18010146 (PMC7794962; doi:10.3390/ijerph18010146)
Supplement: Supplementary file 1 [file ijerph-18-00146-s001.pdf]

International Journal of
Environmental Research and Public Health

MDPI

Supplementary material

Iron Oxide Particles Alter Bacterial Uptake and the LPS-Induced Inflammatory Response in Macrophages

Materials and Methods – Supplement

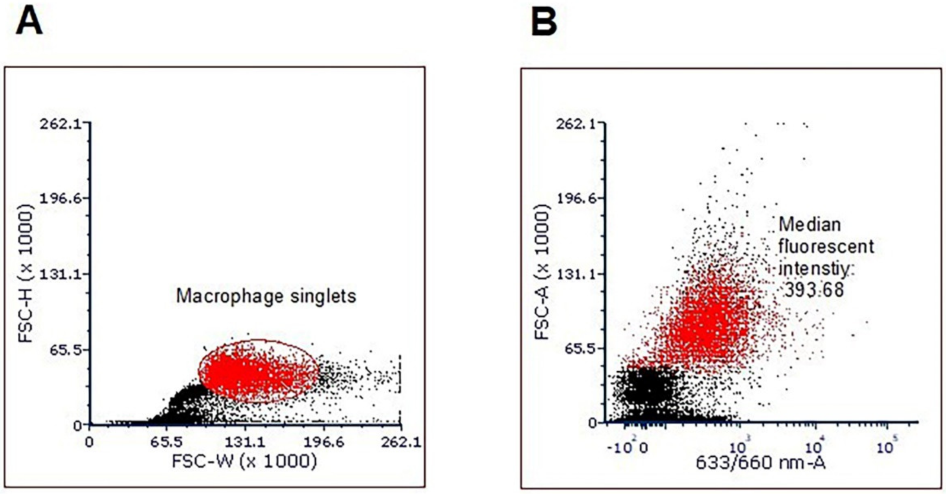

Figure S1. Example flow cytometry analysis of peripheral blood mononuclear cell (PBMC)-derived macrophages phagocytosing NTHi. PBMCs were collected from healthy adults, isolated using density gradient centrifugation and differentiated with GM-CSF. Compensation was performed using single colour stained cells, bacteria, and bacteria-free particle controls with macrophages. Cell populations were distinguished based on forward scatter (FSC) height and width given the distinct increase in size of macrophages compared to cell debris, non-differentiated monocytes and extracellular bacteria that remained after washing (A: FSC-H/FSC-W). Given the non-bimodal nature of phagocytosis, intracellular bacteria were quantified using the median fluorescent intensity rather than as a positive population percentage (B: FSC-A/ 633/660nm-A represents the median fluorescent intensity of macrophages exposed to 633/660nm, Far Red corresponding to stained NTHi).

Results—Supplement

Human primary peripheral blood mononuclear cells (PBMCs) were differentiated into macrophages and exposed to 0 or 50 μg/mL of haematite, magnetite or silica. Twenty-four hours later, cytotoxicity was assessed by lactate dehydrogenase (LDH) production. Quartz induced a significant increase in LDH production when compared to control (p = 0.001) whereas the iron oxides had no effect ($p > 0.47$) (Figure S1).

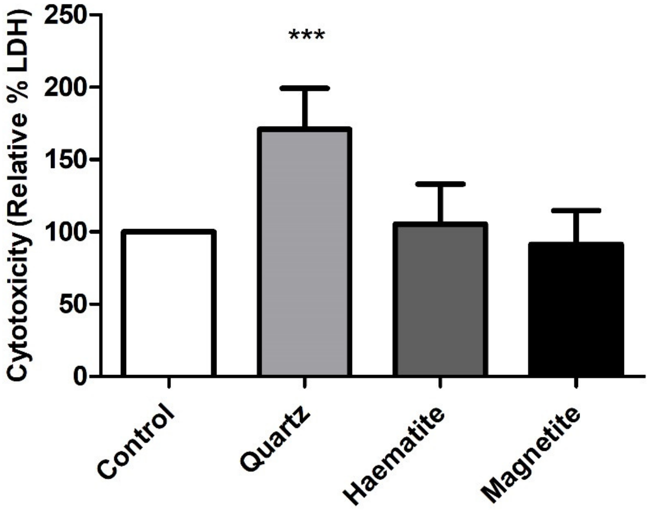

Figure S2. Lactate dehydrogenase (LDH) in the supernatant of PBMC-derived macrophages exposed to quartz, haematite or magnetite for 24 h. LDH data are represented as a relative percentage increase in optical density value compared to the control (100%). Data are presented as mean (SD) from 6 independent experiments. *** indicates $p < 0.001$ versus control.
